# Interaction of antiphospholipid antibodies with endothelial cells in antiphospholipid syndrome

**DOI:** 10.3389/fimmu.2024.1361519

**Published:** 2024-07-09

**Authors:** Weimin Feng, Jiao Qiao, Yuan Tan, Qi Liu, Qingchen Wang, Boxin Yang, Shuo Yang, Liyan Cui

**Affiliations:** ^1^ Department of Laboratory Medicine, Peking University Third Hospital, Beijing, China; ^2^ Institute of Medical Technology, Health Science Centre, Peking University, Beijing, China

**Keywords:** antiphospholipid syndrome, endothelial cells, receptors, intracellular pathways, potential targets for therapy

## Abstract

Antiphospholipid syndrome (APS) is an autoimmune disease with arteriovenous thrombosis and recurrent miscarriages as the main clinical manifestations. Due to the complexity of its mechanisms and the diversity of its manifestations, its diagnosis and treatment remain challenging issues. Antiphospholipid antibodies (aPL) not only serve as crucial “biomarkers” in diagnosing APS but also act as the “culprits” of the disease. Endothelial cells (ECs), as one of the core target cells of aPL, bridge the gap between the molecular level of these antibodies and the tissue and organ level of pathological changes. A more in-depth exploration of the relationship between ECs and the pathogenesis of APS holds the potential for significant advancements in the precise diagnosis, classification, and therapy of APS. Many researchers have highlighted the vital involvement of ECs in APS and the underlying mechanisms governing their functionality. Through extensive *in vitro* and *in vivo* experiments, they have identified multiple aPL receptors on the EC membrane and various intracellular pathways. This article furnishes a comprehensive overview and summary of these receptors and signaling pathways, offering prospective targets for APS therapy.

## 1 Antiphospholipid syndrome

Antiphospholipid syndrome (APS) is a relatively rare autoimmune disorder, with an estimated global prevalence of around 50 cases per 100,000 people, the annual incidence is approximately 1–2 cases per 100,000 individuals (1, 2). APS is linked to diverse antiphospholipid antibodies (aPL) present in patients. Classical aPL include anti-β2 glycoprotein-I antibody (anti-β2GPI antibodies), anticardiolipin antibody (aCL), and lupus anticoagulant (LA) (3). There are also some non-classical antibodies, which are called “non-criteria” antibodies, closely related to APS, such as anti-vimentin/cardiolipin complex, anti-PS/PT antibodies (4). Initially, these antibodies were considered only as diagnostic markers of this disease, a concept that still persists in the current diagnostic guidelines (5). However, subsequent extensive animal experiments and clinical studies have revealed that aPL are not merely biomarkers but play a crucial role in the pathogenic mechanism of APS (6, 7). In the past decades, the Sydney Criteria established in 2006 have been widely recognized as the diagnostic standard for APS, requiring at least one clinical symptom and one positive laboratory test result for a diagnosis of APS. In 2023, new guidelines were proposed by ACR/EULAR, the entry criteria for APS are similar to previous one: aPL-related clinical indicators and positive aPL test within three years. However, it has also been updated to reflect recent advancements, the ACR/EULAR APS classification criteria divide clinical manifestations into six domains: macrovascular venous thromboembolism, macrovascular arterial thrombosis, microvascular involvement, obstetric complications, cardiac valve pathology, and hematologic abnormalities. Laboratory criteria are categorized into three domains: LA, aCL, and anti-β2GPI antibodies. Each clinical and laboratory criterion is assigned a specific weight based on clinical evidence. A diagnosis of APS can be confirmed when both the clinical and laboratory scores reach a threshold of three points each. The new criteria have shown excellent validation results in the population (8, 9). However, the presence of classical aPL is not the gold standard for the manifestation of APS symptoms. The concept of seronegative APS was first introduced in 2003 by Hughes et al., who described patients exhibiting clinical symptoms such as stroke and recurrent miscarriages, yet consistently testing negative for aPL (10). Over time, similar concepts have emerged to describe this same group of patients (11). Due to the unique nature of its definition, diagnosing seronegative APS is typically exclusionary, presenting significant challenges for clinical differentiation. The term ‘seronegative’ refers specifically to the absence of classical aPL in the serum. Consequently, various “non-criteria” antibodies have garnered attention for their potential role in the pathogenesis of APS. These commonly studied non-criteria antibodies include anti-prothrombin/phosphatidylserine antibodies, anti-vimentin antibodies, anti-AnnA2 antibodies, anti-AnnA5 antibodies, anti-phosphatidylethanolamine antibodies, some IgA isotypes of traditional antibodies, and traditional aPLs detectable only by more sensitive methods (12–14). These antibodies either target similar sites as traditional aPL, exerting the same effects, or they act on other molecules within various cell activation pathways, leading to symptoms akin to those of typical APS. Numerous clinical studies and epidemiological data have shown that the prevalence of these antibodies is significantly higher in APS and seronegative APS patients, suggesting their positivity holds diagnostic significance for seronegative APS and warrants further investigation. Nevertheless, the diversity of detection techniques for non-criteria antibodies and the inclusion of these antibodies in the diagnosis of APS or seronegative APS still lack specificity and sensitivity.

Despite recent advancements in diagnosing APS, the pathogenesis of this disease remains elusive. The prevailing “two-hit hypothesis” partially explains the mechanisms behind it, but the more intricate signaling pathways underlying this disease require further investigation. In clinical practice, APS is predominantly characterized by thrombosis in both arteries and veins, as well as recurrent miscarriages. Additionally, it presents with various other clinical manifestations such as livedo reticularis and headaches (15). Consistent with its clinical symptoms involving multiple tissues and organs, several cell types including endothelial cells (ECs), neutrophils, monocytes, platelets and trophoblasts, serve as significant targets for aPL and are integral to these clinical features. Consequently, understanding the molecular-level interactions of aPL with each cellular type is imperative for advancing both the diagnosis and therapeutic approaches for this condition.

## 2 Endothelial cells

ECs are among the primary target cells stimulated by aPL, and their involvement is intimately linked to the clinical symptoms observed in APS, such as hypercoagulability and venous and arterial thrombosis. ECs form a cobblestone-like layer lining the inner walls of blood vessels throughout the body, serving as central players in maintaining cardiovascular homeostasis. They function as a semi-selective protective barrier separating the bloodstream from the vascular wall, facilitating the transport of oxygen and nutrients, regulating blood flow, and maintaining tissue homeostasis (16). Additionally, they possess endocrine functions, secreting various cytokines and tissue factors (TF), such as IL-1, IL-6, VEGF, VCAM-1, and ICAM-1, which are indispensable in regulating immune cell functions and promoting angiogenesis. Among the multiple functions of ECs, two are particularly relevant to the pathogenesis of APS. First, ECs control thrombosis. Under physiological conditions, in coordination with vascular smooth muscle cells, they can freely regulate vascular dilation and contraction. In the process of regulating vasodilation, the two most important molecules are nitric oxide (NO) and prostaglandin I2 (PGI2). NO is an endothelial-derived free radical gas that can be released in response to stimuli like angiotensin II, acetylcholine, histamine, and bradykinin, increasing cGMP levels in smooth muscle cells. PGI2, a unique metabolite of arachidonic acid in ECs, can induce vasodilation. Both NO and PGI2 also inhibit platelet aggregation (17–19). Similarly, various endothelial cell-derived molecules can constrict blood vessels via different mechanisms, such as angiotensin II (Ang II), thromboxane A2, and endothelin-1 (20). In a resting state, ECs’ contact with blood does not lead to platelet aggregation. This is not only due to NO and PGI2 but also because ECs release several substances, including antithrombin III, tissue factor pathway inhibitor (TFPI), thrombomodulin (TM), which can block the coagulation process, and t-PA and u-PA, which effectively promote fibrinolysis, preventing thrombus formation. In summary, the regulation of the thrombus formation process by ECs is both complex and critical. ECs release various factors that play key roles in maintaining the balance between coagulation and fibrinolysis. When ECs are stimulated or damaged, this delicate balance can be disrupted, leading to abnormal coagulation processes and ultimately resulting in thrombosis (21–25). Secondly, ECs actively take part in both adaptive and innate immune responses, possessing functions such as pathogen associated molecular patterns (PAMPs)-, and danger associated molecular patterns (DAMPs)-sensing, anti-inflammatory, cytokine release (26). ECs play an important mediating role in coordinating and supporting the body’s response to inflammation by regulating blood flow, white blood cell transport and cell-cell interactions. During acute inflammation, ECs can elevate blood flow, increase leakage of plasma proteins into the tissue. This process helps promote the binding and activation of neutrophils and other leukocytes, which in turn directs their exportation to the site of inflammation. In the context of chronic inflammation, ECs react to vascular growth factors, facilitating the development of new blood vessels required for sustaining inflammatory tissues (27).

Considering that APS primarily presents with thrombosis as its main symptom, the relationship between APS and ECs has always been a research hotspot. Research findings indicate that aPL can trigger the activation of ECs in APS patients, resulting in an elevated release of TF, inflammatory factors, and adhesion molecules, for instance, ICAM-1, VCAM-1 and E-selectin. This induction is associated with the occurrence of thrombosis and other symptoms (15, 28–34). The established involvement of ECs in the pathogenesis of APS suggests that they could emerge as promising therapeutic targets for the condition. Exploring the interplay between aPL and ECs can not only unveil the fundamental nature of the disease but also foster advancements in disease treatments.

## 3 Receptors on endothelial cells

Initially, there was a prevalent belief that aPL directly bound to anionic phospholipids on the cell membrane. However, subsequent research has shown the involvement of multiple co-receptors in this binding process (35–37). The majority of these aPL, in fact, target phospholipid-binding proteins, with β2-glycoprotein I (β2GPI) being the primary antigen of interest (7, 38, 39). β2GPI is a glycoprotein weighing 48 kDa, also known as apolipoprotein H, composed of five domains, encompassing four regular domains and one atypical domain, resembling a fishhook. β2GPI has multiple functions in the body, such as reducing triglyceride levels and increasing the enzymatic activity of lipoprotein lipase. Most importantly, it plays a crucial role in the regulation of the coagulation process (40–43). N Del Papa and colleagues demonstrated through experiments with HUVECs that human β2GPI binds to ECs through lysine residue clusters which are responsible for binding to anionic phospholipids, providing epitopes for anti-β2GPI antibodies (44). Currently, there is widespread acceptance that when anti-β2GPI antibodies exert their effects, they always simultaneously bind to domain I of two β2GPI molecules, forming complexes. Subsequently, the complex’s domain V specifically attaches to cell surface receptors, with dimeric β2GPI exhibiting significantly higher affinity for ECs than monomeric β2GPI (40). However, the question of how complexes formed by β2GPI and anti-β2GPI antibodies interact with the EC membrane has been a subject of ongoing debate. In research on EC surface receptors over the past few decades, molecules identified to mediate this process include Toll-like receptors (TLRs), apoER2, and other components (**Table 1**).

**Table 1:** The studies on endothelial cell receptors that mediate signals triggered by antiphospholipid antibodies.

Receptors	*In vivo*/*In vitro*	Year	Author	Refs
TLRs	*in vitro*	2003	Elena Raschi et al.	(45)
TLR4	*in vivo*	2007	Silvia S Pierangeli et al.	(46)
	*in vivo*	2016	P Laplante et al.	(47)
	*in vivo*	2019	Meiyun Wang et al.	(48)
	*in vitro*	2021	Guiting Zhang et al.	(49)
TLR2, CD14	*in vitro*	2011	Nathalie Satta et al.	(50)
TLR2	*in vitro*	2010	Jean-Eric Alard et al.	(51)
ApoER2	*in vivo*	2011	Sangeetha Ramesh et al.	(52)
	*in vivo*	2011	Zurina Romay-Penabad et al.	(53)
	*in vivo*	2014	Victoria Ulrich et al.	(54)
	*in vivo*	2018	Sacharidou et al.	(55)
ApoER2, Lipid rafts	*in vitro*	2023	Gloria Riitano et al.	(56)
EPCR	*in vivo*, *in vitro*	2021	Nadine Müller-Calleja et al.	(57)
Annexin II	*in vitro*	2000	K Ma et al.	(58)
	*in vitro*	2005	Jianwei Zhang et al.	(59)
	*in vitro*	2006	Gabriela Cesarman-Maus et al.	(60)
	*In vivo*, *in vitro*	2009	Zurina Romay-Penabad et al.	(61)
Annexin II, TLR4	*in vitro*	2014	MO Borghi et al.	(62)
Annexin II, TLR4, calreticulin, nucleolin	*in vitro*	2012	Kristi L Allen et al.	(63)
LRP6, PAR-2	*in vitro*	2022	Gloria Riitano et al.	(64)

### 3.1 TLRs

The TLRs are constituted by a cluster of type I transmembrane proteins, which function as the innate immune system’s vigilant eyes, actively monitoring and discerning diverse molecular patterns associated with diseases. They stand as the primary defense barrier, essential for the body’s resistance against infectious diseases. Moreover, TLRs play a crucial role in recognizing and regulating processes within the adaptive immune system. They are always found on the membrane of ECs, dendritic cells, macrophages and other cells with special structural features including a leucine-rich repeat (LRR) segment facilitating the recognition of PAMP/DAMP, transmembrane helices and a TIR domain that is responsible for initiating downstream signaling cascade (65–67). Elena Raschi and colleagues discovered in 2003 through cellular experiments that TLRs are involved in the process of anti-β2GPI antibodies activating ECs (45). Among diversiform TLRs, TLR4 and TLR2 are the most relevant to the pathogenic signaling pathways in APS. Various endogenous molecules, such as glycoproteins, phospholipids and intracellular peptides, have the potential to activate TLR4, and TLR4 can mediate inflammatory responses through multiple pro-inflammatory mediators (68). In *in vivo* experiment comparing mice that do not respond to LPS (LPS-/-) with those that respond to LPS (LPS+/+), it was found that aPL induced more significant thrombosis, elevated adhesion of leukocytes to ECs, and higher plasma TF activity in LPS+/+ mice. Because the signaling induced by LPS is transmitted through TLR4, the experimental results illustrated that TLR4 participates in the stimulation of aPL on ECs in the body (46). Subsequent studies have confirmed the role of TLR4 in both trophoblasts and monocytes (69–72). In recent years, its role in ECs has also been validated both *in vivo* and *in vitro*. When anti-β2GPI antibodies were used to stimulate either mice or cultured ECs, there was a marked promotion of thrombosis and inflammation. However, these effects were abolished in TLR4-defective mice or when TLR4 was blocked (47–49). However, the receptor role of TLR4 has also been questioned. Nathalie Satta et al. found that anti-TLR2 antibodies, rather than anti-TLR4 antibodies, inhibited the activation of HUVECs and monocytes induced by aPL. Additionally, pre-treating HUVECs with TNF increased the expression of TLR2 but had no effect on TLR4, leading to an enhanced inflammatory reaction to aPL. Thus, it is suggested that aPL do not activate monocytes and ECs through interaction with TLR4 but rather through another member of the TLR family, TLR2 (50). TLR2 is highly expressed in immune cells, especially innate immune cells, and its function in non-hematopoietic cells like ECs, has gradually received attention (73). Although this research has cast doubt on TLR4, it has confirmed the role of TLR2 in fibroblasts by the same team (74) and in ECs by other researchers (51). Furthermore, some researchers believe that TLR7 and TLR8 on the cell membrane play crucial roles in this process, but their roles in ECs warrant further investigation (75–77).

### 3.2 ApoER2

Apolipoprotein E receptor 2 (ApoER2), also known as LDL receptor-related protein 8 (LRP8), is a member of the low-density lipoprotein receptor family. It is a transmembrane endocytic receptor protein widely present on cell membranes. Its configuration encompasses five functional domains that exhibit structural similarities to the receptors of low-density lipoprotein (LDL) and very low-density lipoprotein (VLDL), with high affinity for ApoE (78, 79). Additionally, ApoER2 plays a role in cell signaling (80). Considered that ApoER2’, a splice variant of ApoER2, is a receptor on platelet that several labs have reported can bind to β2GPI dimers and mediate subsequent cell responses (81, 82). Furthermore, ApoER2 itself regulates the coagulation process. Therefore, the potential role of ApoER2 on other cells is also under investigation (83). Sangeetha Ramesh and colleagues found that in ApoER2+/+ mice, aPL enhanced thrombosis, leading to a shortened time to complete vascular occlusion. Conversely, the presence of aPL did not affect thrombosis in ApoER2-/- mice (52). Models using ApoER2+/+ and ApoER2-/- mice have been employed in multiple studies, successfully demonstrating that ApoER2 is a critical receptor in the pathogenic mechanism of aPL. For instance, in wild-type mice, aPL increased vascular TF activity, thrombosis, and activated monocytes, but these adverse outcomes were significantly reduced in ApoER2-/- mice (53). Compared to ApoER2+/+ mice, ApoER2-/- mice showed reduced inhibition of endothelial regeneration by aPL. This was corroborated by cell experiments where siRNA knockdown of ApoER2 in ECs restored the migration ability inhibited by aPL, proving that aPL impairs endothelial repair involving ApoER2-mediated β2GPI recognition (54).

When discussing the LDL receptor family as aPL receptors, the concept of “lipid rafts” must be introduced. Lipid rafts are lipid microdomains in the cell membrane enriched in sphingolipids and cholesterol, serving as platforms for protein attachment and signal transduction (84). As early as 2007, studies described that annexin II and TLR4 in lipid rafts on monocyte membranes can promote inflammation in APS (72). In recent years, the role of lipid rafts on EC membranes in the context of aPL has also been updated. One hypothesis is that the receptors for anti-β2GPI antibodies are likely located in lipid rafts on the EC membrane, involving LRP6 and its co-receptor PAR-2. Upon binding of these three, the complex initiates the phosphorylation of β-catenin, ultimately resulting in increased TF expression (64). Another study, based on Sacharidou’s experiments, demonstrated that ApoER2, with the assistance of Disabled-2 (Dab2) and Src homology 2 domain-containing protein 1 (SHC1), can form complexes to receive aPL (55). Thus, it is reasonable to further consider the involvement of lipid rafts in this process. Gloria Riitano and colleagues used MβCD to disrupt lipid rafts on EC membranes, significantly reducing LRP8-mediated signal transduction (56). These results collectively indicate that ApoER2 is an important aPL receptor, mediating ECs activation. It also supports that the structure of lipid rafts holds a special position in the pathogenesis of APS. The intracellular signaling pathways mediated by ApoER2 as a receptor will be detailed in the next section. If the integrity of these rafts is disrupted, it is likely to interrupt the signal transmission. Therefore, lipid rafts could be considered as a therapeutic target for APS, warranting further investigation and attention.

### 3.3 EPCR

Another β2GPI/anti-β2GPI antibodies complexes receptor related to APS is endothelial protein C receptor (EPCR), a membrane-bound protein expressed in several kinds of cells, contributing to placental development and anticoagulant system. EPCR is of significant importance in this context, as it enhances the activation of protein C (PC) by the thrombin-thrombomodulin complex, resulting in a 20-fold increase (85, 86). In APS patients exhibiting obstetric symptoms, researchers have detected significantly higher levels of IgM anti-EPCR antibodies compared to the normal population. These antibodies can inhibit the generation of activated protein C on the endothelium and are an independent risk factor for fetal loss (87). This is consistent with earlier research findings, suggesting that aPL-induced resistance to activated protein C might be a potential mechanism underlying thrombotic event (88, 89). Experimental evidence in mouse models has shown that EPCR contributes to the pathological outcomes induced by aPL. aPL can bind to EPCR on the cell membrane, the complex formed by EPCR and lysobisphosphatidic acid (LBPA) undergoes accelerated endocytosis in the presence of these antibodies. This process results in a substantial presence of antibodies in the late endosomes of ECs, inducing thrombin-PAR1 signaling, which translocates acid sphingomyelinase (ASM) and activates cell (57, 90, 91). Additionally, clinical population data have indicated that different haplotypes of EPCR can impact the symptoms of APS patients. Specifically, it has been demonstrated that the H1 haplotype is associated with a diminished likelihood of arterial thrombosis in these patients (92). A recent study on hospitalized COVID-19 patients also found that the levels of lipid-binding aPL IgG were higher in these patients compared to the healthy population, and these immunoglobulins could bind to the EPCR-LBPA complex, producing a pathological mechanism similar to APS (93).

### 3.4 Annexin II

Annexins constitute a multigene family of Ca2+-regulated proteins known for their distinctive Ca2+ and membrane-binding module, referred to as the annexin core domain. The annexin core domain allows Ca2+-bound annexins to attach to membranes containing negatively charged phospholipids at the periphery, which plays essential roles in regulating cell growth and signal transduction pathways (94, 95). As important regulatory proteins, Annexin II was identified as an important aPL receptor on EC membranes as early as 2000. In their study, Keying Ma et al. used radiolabeling methods to confirm the presence of β2GPI-binding proteins on the surface of ECs, subsequently isolating and purifying the corresponding protein, which they identified as Annexin II (58). Since then, the role of Annexin II in APS has garnered significant attention. Multiple cell and animal experiments have further elucidated that the stimulation mediated by anti-β2GPI antibodies through Annexin II receptors is a primary cause of ECs activation and thrombosis induction in APS. In ECs, anti-Annexin II antibodies can induce similar cellular activation as anti-β2GPI antibodies (59). In mice deficient in Annexin II, the size of anti-phospholipid antibody-induced thrombi and the activity of TF are significantly reduced (61), supporting these findings. A clinical study based on a Mexican population also indicated that Annexin II is meaningful for both APS clinical diagnosis and mechanistic research, as the positive rate of anti-Annexin II antibodies in the serum of APS and SLE patients was significantly higher than that in healthy individuals and patients without thrombosis (60). However, there are divergent views regarding Annexin II. Considering that Annexin II is not a transmembrane protein and lacks intracellular signaling pathways, questions arise regarding how it facilitates signal transduction between aPL and ECs. Subsequent research has suggested that Annexin II might only be part of the aPL receptor complex on ECs, possibly forming co-receptors with TLR2 or TLR4 to mediate this stimulation (45, 63, 96). One notable study by Kristi L. Allen et al. identified a complex involving Annexin A2, TLR4, calreticulin, and nucleolin that performs this function on ECs (63). Besides Annexin II, another member of the annexin family, Annexin V, also plays a significant role in the pathogenesis and progression of APS. However, unlike Annexin II, Annexin V is not a receptor for aPL on ECs. Instead, its presence can prevent aPL from binding to phospholipids on the cell membrane. Only when the protective layer formed by Annexin V on the cell surface is disrupted can aPL interact with ECs.

### 3.5 Other accessory molecules

Apart from the previously mentioned potential independent receptors, there are also some co-receptors that serve as accessory molecules in the EC activation triggered by aPL. Other examples include CD14, calreticulin and nucleolin, the former can serve as co-receptors for TLR2 and TLR4, while the latter two can form complexes with Annexin II, TLR4, which are present on the cytomembrane of ECs, and mediate EC responses to β2GPI and anti-β2GPI antibodies (50, 63, 97). Similarly, the specific roles of these molecules are also subject to debate.

## 4 Signaling pathways in endothelial cells

**Figure 1:**
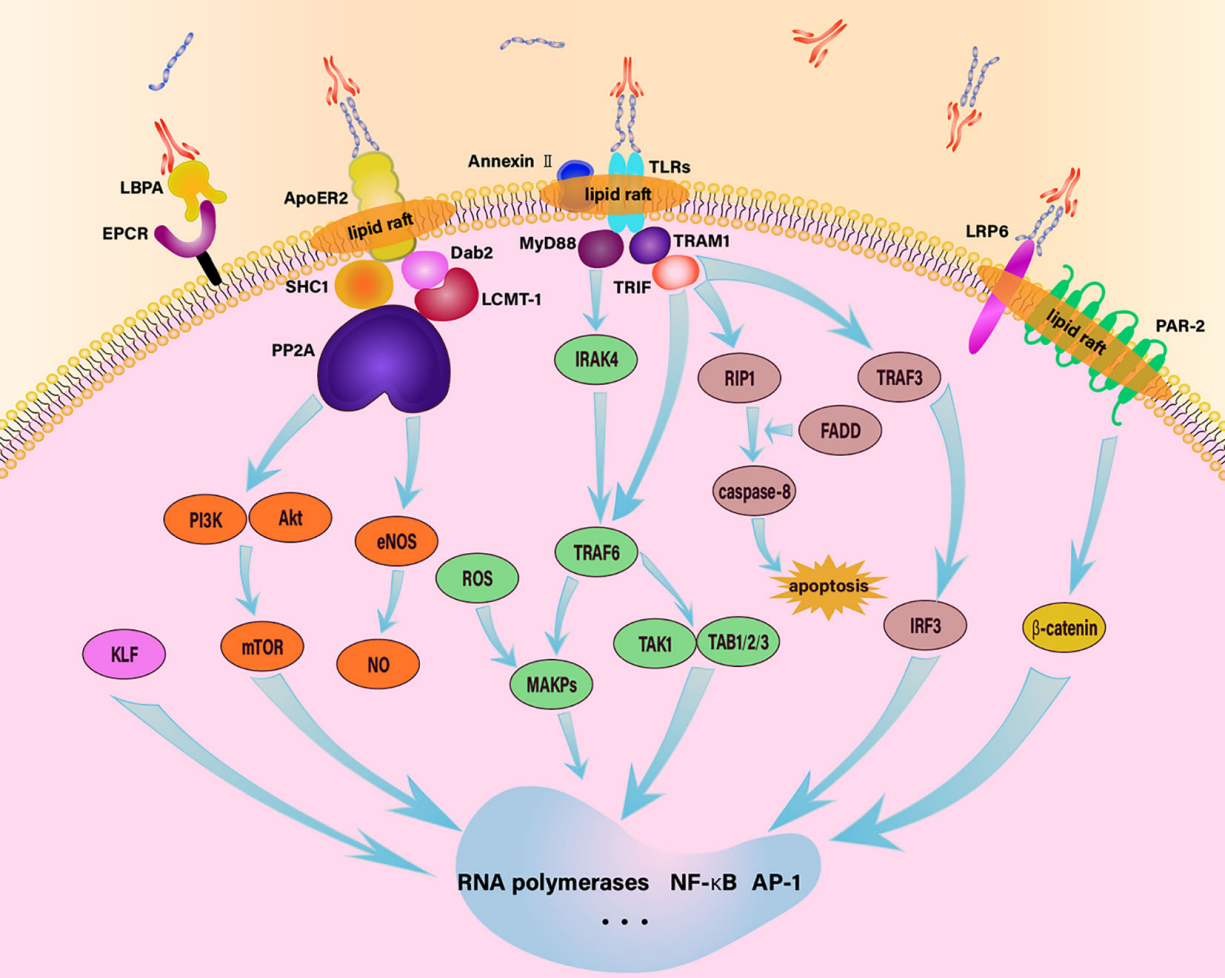
Schematic diagram of endothelial intracellular signaling pathway in response to aPL. aPL activates ECs through various signaling pathways, including TLR4/MyD88, TLR4/TRIF, EPCR/LBPA, LRP6/PAR-2, MAPK, apoER2-Dab2-SHC1, and mTOR, thereby regulating downstream cellular activities.

The reception of stimulating signals by these receptors is just the first step in initiating EC responses. These signals need to be transferred from the extracellular space to the intracellular environment, where they regulate the gene expression and release of relevant molecules through a complex network of signals, contributing to the development of inflammation, thrombosis, and other diseases (**Figure 1**). The process involves numerous pathways and is influenced by various molecules. Currently, several signaling pathways have been identified with relative clarity, including the following.

### 4.1 Pathway one: TLR4/MyD88 pathway

β2GPI/anti-β2GPI antibodies complexes activate ECs through the TLR4/MyD88 pathway (45, 46, 98). TLRs signal through five main proteins, including TIR-domain-containing adaptor protein inducing IFNβ (TRIF), myeloid differentiation factor 88 (MyD88), MyD88-adaptor-like protein (MAL), sterile α- and armadillo-motif-containing protein (SARM) and TRIF-related adaptor molecule (TRAM). TLR adaptors are protein molecules featuring Toll/IL-1 receptor (TIR) domains that engage with TLRs through TIR-TIR domain interactions, which is responsible for the propagation of downstream signaling. These five proteins play different roles in the TLR4-mediated signaling process (99). MyD88 serves as a typical adaptor for downstream inflammatory responses of TLRs and IL-1 receptors. It is essentially a cytoplasmic soluble protein with three functional regions in its structure: the N-terminal death domain (DD), an intermediate region, and the TIR domain. The DD can mediate interactions between proteins that have homotypic DD, and the TIR domain is similar to the cytoplasmic region of the IL-1 receptor, transmitting signals by recruiting other TIR domain-containing proteins (100). Mal, alternatively recognized as Toll/IL-1 receptor domain containing adaptor protein (TIRAP), is a protein essential for the signaling of TLR2 and TLR4. Initially, it was thought to serve as a bridge exclusively to MyD88. However, with the discovery of various “MyD88 bridging-independent” functions of Mal, it is now widely recognized for its diversified functions (101). TRIF contains a globular helical N-terminal domain, a TIR domain, a TRAF6- and a TRAF2-binding motif and a C-terminal RHIM. The significance of TRIF-dependent signaling in host defense is evident (102). TRAM shares a structural similarity with Mal, and it has been demonstrated to be vital in the TLR4 signaling pathway in both TRAM-deficient mouse and cellular models (103). SARM is a member of the TLR adapter family, encoding a protein featuring armadillo motif (ARM) and sterile alpha motif (SAM) domains. SARM negatively regulates MyD88 and TRIF-dependent TLR signaling in the immune response (104–106).

After β2GPI/anti-β2GPI antibodies complexes stimulate EC membrane TLR4 receptors, MyD88 is enlisted to bind with TLR4, initiating the activation of IRAK4, which enables the binding and phosphorylation of IRAK1. Once IRAK1 is phosphorylated, it can recruit TRAF6, which subsequently activates TAK1/TAB1/2/3 and MAPKs. Downstream of these pathways, the activation of NF-κB and transcription factor AP-1 results in the expression of inflammatory genes in ECs (96, 107). SARM operates as an inhibitory factor within the signaling cascade mediated by MyD88, exerting negative regulatory control, the SARM-TIR domain primarily interacts with MyD88 and TRIF through its structural BB-loop (108, 109).

### 4.2 Pathway two: TLR4/TRIF pathway

This pathway primarily involves the proteins TRIF and TRAM1. TRAM1 acts as an intermediary, linking TLR4 with TRIF, and upon TLR4 signaling, TRIF can bind to both TRAF6 and RIP1. These interactions lead to the activation of NF-κB via two separate pathways, promoting the expression of inflammatory genes. Additionally, RIP1 can induce apoptosis through a mechanism involving caspase-8 activation via FADD. TRIF can also activate TRAF3, which recruits TANK, TBK1, and IKKE, consequently, these activities trigger the activation of IRF3 and the subsequent expression of multiple genes (98, 99). Similar to the TLR4/MyD88 pathway, the TLR4/TRIF pathway is also negatively regulated by SARM.

### 4.3 Pathway three: MAPK pathway

Mitogen-Activated Protein Kinases (MAPKs) are a group of serine-threonine protein kinases activated by various extracellular and intracellular stimuli such as cytokines, neurotransmitters, hormones, cellular stress, and cell adhesion. MAPKs serve as important transducers in conveying signals from the cell surface to the nucleus, modulating the activity of pertinent genes (110). The MAPK pathway comprises four main branches, with p38MAPK being one of them. p38MAPK is primarily involved a cascade of kinases that transmit extracellular signals into the cell (111). The functional modulation of ECs in APS is significantly influenced by the p38MAPK pathway, and the regulatory role of MAPKs in ECs has been well established in numerous studies. For example, the experiments of Vega-Ostertag’s team illustrated that the upregulation of TF transcription, as well as the regulation of IL-6 and IL-8 in ECs induced by aPL, is mediated through the phosphorylation of p38MAPK and activation of NF-κB. The experimental results from Simoncini et al. indicate that APS-IgG has the capacity to elevate the levels of VCAM-1 secretion by ECs as well as phosphorylation of p38 MAPK. Inhibition of p38MAPK with SB203580 significantly reduced THP-1 adhesion to ECs *in vitro*, thrombus size, the attraction of ECs to leukocytes, TF activity in carotid arteries, and the level of VCAM-1 expression (112–115). After treatment with β2GPI/anti-β2GPI antibodies complexes, there is an increase in the expression of TRAF6. TRAF6, in turn, triggers the activation of MEK3 and MEK6, both of which serve as kinases positioned upstream of p38 and JNK, this activation occurs through the activation of MAPK. aPL also induce the generation of reactive oxygen species (ROS) in ECs, then ROS, acting as second messengers, activate p38MAPK and regulate ECs (112, 116).

### 4.4 Pathway four: apoER2-Dab2-SHC1 pathway

Additionally, some pathways are mediated by ApoER2. Under the effect of anti-β2GPI antibodies, β2GPI forms dimers and interacts with apoER2, forming an apoER2-Dab2-SHC1 complex within the ECs, this complex is capable of linking with PP2A. Leucine methyltransferase-1 can methylate the catalytic subunit of PP2A at L309, a process that is accelerated in the presence of Dab2 recruited onto the apoER2 NPXY motif. Simultaneously, the scaffolding subunit of PP2A can also be recruited to the proline-rich C-terminus of apoER2 bySHC1. Facilitating the assembly of the PP2A heterotrimer initiated by aPL, two unique regulatory PP2A subunits, Bδ and B′α, play different roles in recruiting Akt and eNOS because of substrate specificity. This recruitment leads to their inhibitory dephosphorylation and reduced NO levels, ultimately contributing to thrombosis (55).

### 4.5 Pathway five: mTOR pathway

In a study by Canaud et al. on renal ECs and vascular ECs, mTOR, an atypical serine/threonine protein kinase, is found to have a substantial regulatory impact (117). This enzyme is composed of two complexes, namely mTORC1 and mTORC2, each regulated differently and with distinct functions. mTORC1 predominantly regulates cell growth and metabolism and is sensitive to rapamycin, while mTORC2 primarily modulates cell survival, proliferation, and cytoskeletal remodeling and is insensitive to rapamycin. It participates in multiple biological processes, including gene transcription, protein translation, and ribosome synthesis, playing a crucial role in cell growth, apoptosis, autophagy, and metabolism (118). In APS, mTOR is primarily involved in the PI3K/Akt/mTOR signaling axis, which is crucial for regulating autophagy (119). However, the relationship between mTOR and autophagy in ECs under aPL stimulation remains ambiguous and sometimes contradictory. Some studies suggest that aPL suppresses autophagy in ECs, leading to endothelial dysfunction and vascular homeostasis disruption (120, 121). Conversely, other studies propose that aPL activates autophagy in ECs (122). Both perspectives acknowledge the role of mTOR in regulating autophagy in ECs. When the β2GPI/anti-β2GPI antibodies complex interacts with its receptors, signal transduction to the intracellular environment occurs. This process involves IRS1 activating PI3K, which phosphorylates PIP2 to generate PIP3. PIP3 can recruit Akt and PDK1 to the plasma membrane. Within the TSC1-TSC2 complex, TSC2 is phosphorylated at multiple sites with the assistance of Akt, leading to the activation of mTORC1. Additionally, Akt can activate mTORC1 by phosphorylating the proline-rich Akt substrate of 40 kDa (PRAS40) (123, 124). Upon receiving upstream signals from the PI3K/Akt pathway, mTOR regulates various signaling pathways that influence cellular translation. Beyond translation, mTOR also modulates protein synthesis by regulating RNA polymerase 1 and 3. This ultimately mediates the effects of aPL on ECs (117, 125, 126). Moreover, Akt downstream signaling can regulate the eNOS molecule, thereby controlling NO production and autophagy (120).

### 4.6 Other pathways

Many studies have explored the intracellular signaling that occurs in ECs upon the action of aPL and how it ultimately results in the release of various procoagulant and inflammatory factors. Apart from the pathways mentioned above, several other molecules and signaling cascades have been demonstrated to contribute to this intricate pathological mechanism, although their upstream and downstream mechanisms remain less clear. For instance, the inhibition of Krüppel-like factor (KLF) expression, in the study conducted by Kristi L. Allen et al, the suppression of KLF expression, supported by β2GPI/anti-β2GPI antibodies interaction, promotes EC activation through the dysregulated activation and transcription of NF-κB, along with the downstream upregulation of pro-thrombotic and pro-inflammatory gene expression (127). Additionally, in Sangeetha Ramesh’s study, the authors found that aPL promoted vascular occlusion in eNOS+/+ mouse models, whereas the addition of aPL had no effect on vascular occlusion time in eNOS-/- mouse models, indicating that the procoagulants effect of aPL is mediated through eNOS. This study also discovered that β2GPI could interact with apoER2, leading to increased PP2A activation, subsequent dephosphorylation of eNOS at S1179, reduced enzyme activity, decreased bioavailable NO, enhanced thrombus formation and elevated adhesion of leukocytes (52).

### 4.7 Signaling pathways and thrombosis

**Figure 2:**
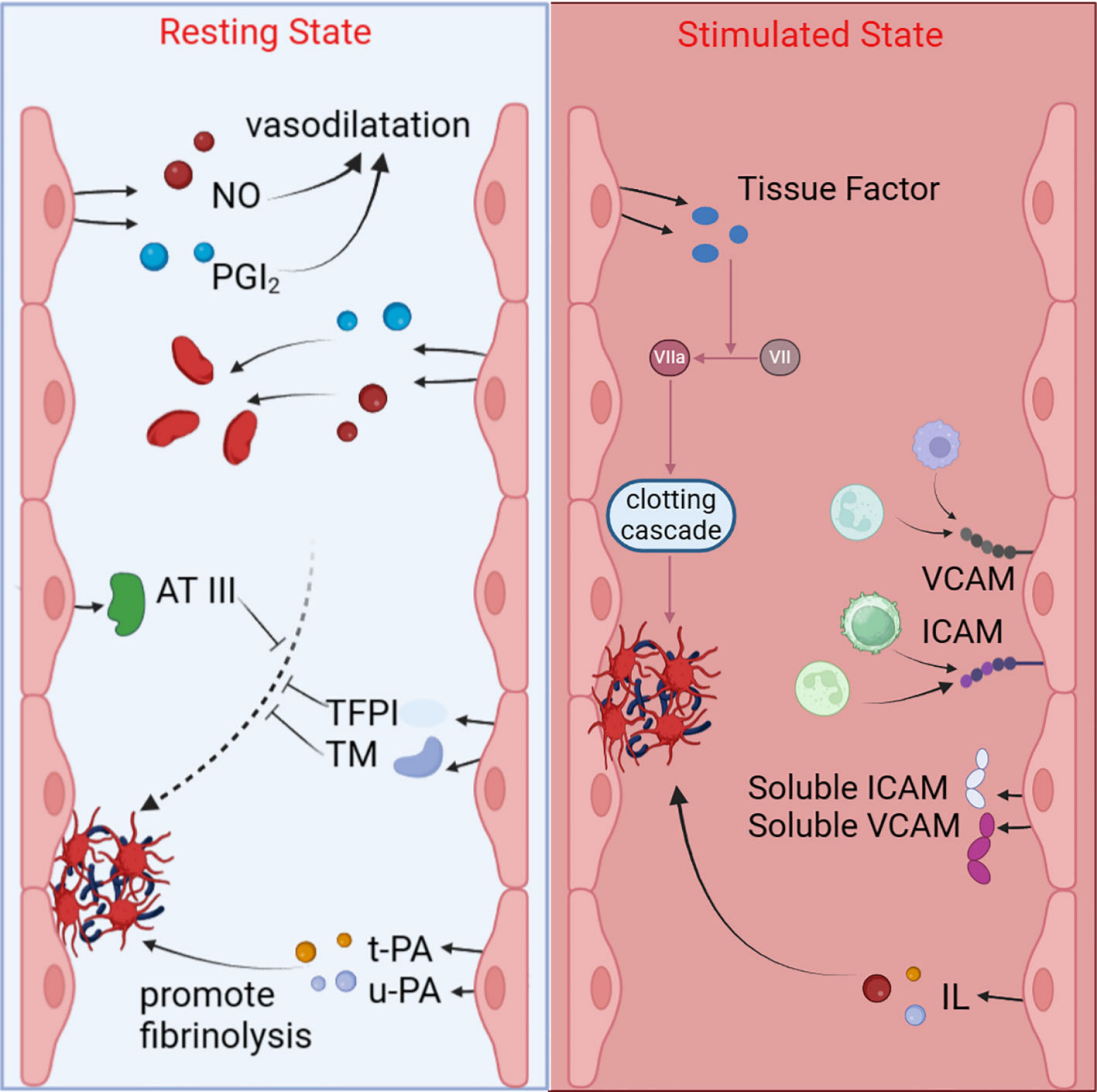
Functions of quiescent and activated endothelial cells. In the quiescent state, ECs release NO and PGI2 to promote vasodilation and inhibit platelet aggregation; they also secrete AT III, TFPI, TM, t-PA, and u-PA to inhibit coagulation and promote fibrinolysis. When stimulated by aPL, ECs promote coagulation and inflammation by releasing substances such as interleukins, ICAM, VCAM, and TF.

The aforementioned signaling pathways convert the external stimuli brought by aPL into various gene expression abnormalities within the EC nucleus, ultimately leading to protein synthesis dysregulation and functional abnormalities in the body. Endothelial dysfunction results in the disruption of the vascular endothelial barrier, exposing the underlying matrix, which facilitates platelet adhesion and aggregation. During the process of ECs apoptosis, the anticoagulant activity on its surface diminishes. Concurrently, dysfunctional ECs secrete a range of pro-coagulant factors, such as TF and von Willebrand factor (vWF), which play core roles in the coagulation cascade, further enhancing the coagulation response. ECs also release various inflammatory mediators, such as interleukins and tumor necrosis factor, which attract and activate more platelets and leukocytes, exacerbating the local inflammatory response. Ultimately, these changes lead to thrombosis, characterized by the formation of insoluble fibrin networks and cell aggregates within the blood vessels, obstructing normal blood flow (128, 129). This not only increases the risk of cardiovascular events, such as myocardial infarction and stroke, but also can result in deep vein thrombosis and pulmonary embolism, leading to severe clinical consequences (**Figure 2**).

## 5 Potential targets for therapy

**Figure 3:**
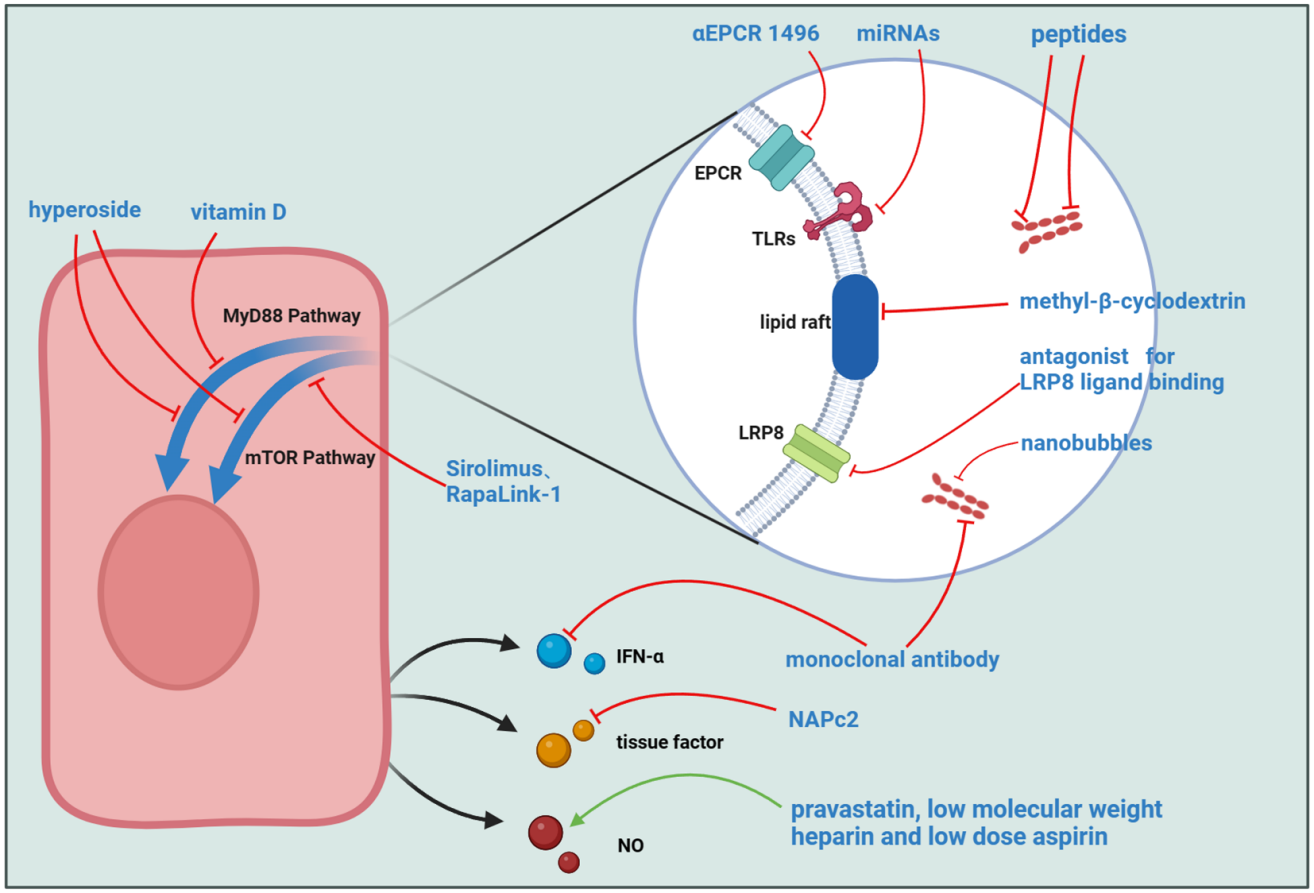
Potential Targets for APS Treatment. Several molecules can interfere with the binding process of aPL to ECs, including αEPCR 1496, miRNAs, peptides targeting domains I and V of β2GPI, methyl-β-cyclodextrin, LRP8 ligand-binding antagonists, monoclonal antibodies, and plasminogen activator-coated nanobubbles. Other molecules act on intracellular signaling pathways, such as Hyperoside, Vitamin D, and mTOR inhibitors like Sirolimus and RapaLink-1. Additionally, some molecules target downstream signal transduction in ECs, including monoclonal antibodies, the TF inhibitor NAPc2, and a combination of pravastatin, low molecular weight heparin, and low dose aspirin.

The current therapeutic paradigm for APS predominantly centers around antithrombotic interventions. Additionally, preventive treatments based on different diagnostic indicators and clinical symptoms, interventions for catastrophic APS, and treatments during pregnancy are employed (130), commonly used medications include low-molecular-weight heparin (LMWH), hydroxychloroquine, among others (131–133). This review delineates the activated pathways in ECs under the influence of aPL, offering potential targets and pharmacological options for clinical APS management (**Table 2**, **Figure 3**). Including inhibition of mTOR-mediated signaling, structural changes of β2GPI, etc., all of which show good performance in *in vitro* and *in vivo* experiments and have great potential for further research (149, 150, 157, 160).

**Table 2:** The potential targets and drugs for APS therapy.

Inhibitors	Targets	Mechanisms	Effects	Refs
Hyperoside	mTOR/S6K, TLR4/MyD88/NF-kB	downregulate the expressions of phosphorylated mTOR, phosphorylated p70S6 Kinase and reduce the levels of MyD88, TLR4, and phosphorylated NF-kB p65.	improved pregnancy outcome, including a lower rate of fetal resorption and increased fetal weight.	(134)
		stimulate the occurrence of cellular autophagy while concurrently hindering the nuclear translocation and phosphorylation and of NF-κB p65	reduced expression of inflammatory factors and endothelial adhesion molecules, such as IL-6, TF, VCAM-1, etc.	(121)
Vitamin D	TLR4/MyD88	inhibit the TLR4/MyD88 signaling pathway, thereby reducing TF release downstream and diminishing ECs activation effects.	limit inflammation and subsequent adverse outcomes in APS pregnancies	(135, 136)
Peptides that target domain V of beta-2-glycoprotein I	domain V of beta-2-glycoprotein I	block Domain V from binding to cell surfaces	decreased β2GPI binding to ECs, diminished interaction between aPL and human trophoblast, and attenuated capacity of aPL to induce thrombosis or fetal loss in murine models	(137, 138)
Peptides that target domain I of beta-2-glycoprotein I	domain I of beta-2-glycoprotein I	block binding of APS-IgG to β2GPI	suppress the thrombotic-promoting capacity of APS-IgG	(139)
monoclonal antibody	beta-2-glycoprotein I	prevent the formation of pathogenic complexes involving relevant antibodies and β2GPI	diminished inhibitory effect of aPL on EC migration, and reversed impairment in reendothelialization caused by aPL	(140)
	beta-2-glycoprotein I	prevents binding to the complement, but has a high affinity with β2GPI	loss of procoagulant and proabortive effects	(141)
	IFN-α	Target IFN, blocking downstream cascading reactions	reduced expression level of IFN and symptom relief	(142–148)
Sirolimus、RapaLink-1(mTOR Inhibitors)	mTOR	promotes autophagy via inhibit mTOR	reduced size of thrombus and lower antibody concentration	(149–154)
miRNAs	TLR-7 and TLR-9	miRNA is an important epigenetic regulatory factor in the mRNA transcription, capable of modulating downstream TLR-mediated IFN generation	reduced IFN-scores, a significant factor influencing both subclinical and clinical manifestations in APS	(155)
TF-inhibitor NAPc2	tissue factor	specific blockade of the TF coagulation initiation complex	diminished prothrombotic effects of aPL and the aPL–induced proinflammatory activation	(156)
αEPCR 1496	EPCR	inhibit the effect of EPCR by binding with it	reduced expression of TNF, F3, IFR8, and GPB6	(93)
plasminogen activator-coated nanobubbles	beta-2-glycoprotein I	rtPA-coated nanobubbles targeting cell-bound β2GPI clear occluded vessels	reduction of new thrombosis, recanalization of occluded blood vessels, and reduction of fibrin deposition	(157)
antagonist for LRP8 ligand binding	LRP8	inhibit the action of LRP8 as a receptor	inhibited phosphorylation of LRP8 and Dab, prevent the overexpression of TF, IL-6 and adhesion molecules	(56, 158)
statins, methyl-β-cyclodextrin	lipid raft	damage the structure of the lipid raft through inhibiting cholesterol synthesis or depleting membrane cholesterol	inhibited phosphorylation of LRP8 and Dab, prevent the overexpression of TF, IL-6 and adhesion molecules	(56, 158)
pravastatin, low molecular weight heparin and low dose aspirin	eNOS/NO	increase eNOS synthesis and activity, resulting in a substantial rise in nitric oxide (NO) production.	improved placental haemodynamics, ameliorated preeclampsia symptoms and improved fetal growth	(159)

Various drug classes exert direct effects on beta-2-glycoprotein I. MBB2, an antibody similar to aPL, can induce thrombosis and adverse pregnancy outcomes *in vivo*. However, a variant antibody lacking the CH2 domain, essential for complement binding and activation, was found to eliminate the pro-coagulant and pro-abortive effects of MBB2. This variant can block the binding of aPL to β2GPI, preventing adverse outcomes in patients (141). Other agents include peptides targeting domains I and V and the monoclonal antibody 1N11. These molecules, upon binding to β2GPI, usurp its functional sites, disrupting its pivotal role in APS pathogenesis and consequentially impeding downstream cellular activation (137–140). Nevertheless, these drugs encounter challenges such as limited half-lives and a dearth of *in vivo* experimental substantiation (135).

There are also several drugs that target aPL action sites or downstream signaling pathways. Considering the pivotal role of the TLR family as receptors for aPL on EC surfaces, constraining their function emerges as a pivotal APS treatment strategy. Hyperoside demonstrates efficacy in mitigating APS symptoms in both *in vivo* and *in vitro* context (121, 134). Its mechanisms encompass the inhibition of TLR4 expression, downregulation of mTOR phosphorylation, and stimulation of cellular autophagy, constituting effective measures for dampening cellular inflammation. In the study conducted by Vandana Gambhir and colleagues, Vitamin D was found to effectively inhibit the TLR4/MyD88 signaling pathway, ultimately mitigating adverse outcomes during pregnancy (136). Remarkably, microRNAs (miRNAs) emerge as potential key players in APS treatment, modulating the TLR-mediated interferon production cascade through epigenetic modifications of pertinent mRNA (155). The integrity of lipid rafts is essential for their role in signal transduction. Given recent studies demonstrating the critical intermediary function of the lipid raft-LRP8 system in the stimulation of ECs by APS, inhibitors targeting this system may alleviate clinical symptoms and serve as potential therapeutic targets for APS. RAP and methyl-β-cyclodextrin (MβCD) are inhibitors of LRP8 and lipid rafts, respectively. Experimental results indicate that these two molecules can almost completely inhibit the phosphorylation of LRP8 and Dab-2 in ECs induced by aPL (56). In a previously mentioned study on EPCR, researchers found that the inflammatory response and pro-coagulant phenotype induced by immunoglobulins in COVID-19 patients could be suppressed by complement factor 3 inhibitor compstatin and inhibitory αEPCR 1496, suggesting these molecules could also be considered for APS treatment (93).

TF is a critical factor in the process of thrombogenesis. Nadine Müller-Calleja and colleagues used NAPc2, an inhibitor of the TF coagulation initiation complex, effectively delaying the progression of APS (156). Furthermore, mTOR inhibitors such as Sirolimus and RapaLink-1 demonstrate efficacy in APS, influencing cellular autophagy and protein synthesis, findings substantiated by experimental and case reports (149–154). The mechanism of triple therapy with pravastatin, low-dose aspirin (LDA) and low molecular weight heparin (LMWH) implicates the pathways activated in ECs post-stimulation by aPL. The combined application of LMWH and LDA induces heightened eNOS levels within ECs, and pravastatin enhances the stability of eNOS mRNA. Consequently, the synergistic use of these three drugs promotes an elevation in NO levels through augmented eNOS levels, ultimately culminating in the amelioration of clinical symptoms (159). Paolo Macor et al. proposed an innovative therapeutic strategy different from the approaches mentioned above. They encapsulated plasminogen activator within nanobubbles and conjugated these nanobubbles with recombinant antibodies targeting β2GPI, achieving highly specific and efficient thrombolytic therapy in APS-prone thrombosis sites (157).

## 6 Discussion

APS is a complex and multifactorial disorder, presenting a challenging topic in understanding its pathogenesis. ECs play an indispensable role in one of the primary clinical manifestations of APS - thrombosis. This article elucidates how ECs interact with aPL in APS patients and transmit these stimulatory signals, it also outlines potential therapeutic targets based on related pathways. Experimental results underscore the significant contribution of ECs to manifestations like thrombosis and inflammation in APS. However, conflicting conclusions arise from different experiments, questioning whether a particular receptor mediates the pathological effects of APS and which branch of a pathway achieves the desired effects. These controversies may stem from variations in antibody types used in experiments, the experimental environment (*in vivo* or *in vitro*), and the intrinsic complexity of APS, where multiple molecules and mechanisms exert diametrically opposed effects in different stages or organs of the disease. It’s essential to recognize that ECs are just one facet of this intricate puzzle.

APS exhibits substantial individual variations in clinical presentations, pathogenic mechanisms. Emphasizing the consideration of individual differences in formulating relevant diagnostic indicators and conducting clinical studies, our indicators and research outcomes need validation in a larger population to ensure broader applicability. Further exploration of the pathological mechanisms of aPL highlights recent findings involving traditional inflammatory signaling pathways and some relatively specific pathways like apoER2-Dab2-SHC1. In addition to established pathways, mechanisms such as multipoint phosphorylation, epigenetic modifications, and cellular processes like autophagy, apoptosis, and ferroptosis are likely closely associated with APS and warrant increased attention. Lastly, current mainstream treatments target symptoms, potentially accompanied by inevitable side effects. In contrast, we underscore the potential of targeted immunotherapy, contingent upon a clearer understanding of the core signaling pathways underlying APS and the identification of suitable target sites.

Modern sequencing and omics technologies, such as single-cell sequencing, efficient tools in immunological and oncological research, and immunome repertoire sequencing, represent promising avenues. Their application can provide deeper biological insights, unraveling the complexities of this disease. These methods aid in characterizing the overall features of cells and molecules related to APS patients while offering higher resolution to finely distinguish the heterogeneity between APS patients and healthy individuals. Simultaneously, rigorous experiments and in-depth molecular studies are imperative to continually advance our understanding of APS and the development of therapeutic approaches.

## Author contributions

WF: Conceptualization, Writing – original draft, Writing – review & editing. JQ: Writing – review & editing. YT: Writing – review & editing. QL: Writing – review & editing. QW: Writing – review & editing. BY: Writing – review & editing. SY: Project administration, Writing – review & editing. LC: Conceptualization, Funding acquisition, Project administration, Writing – review & editing.
